# A fuzzy TOPSIS based analysis toward selection of effective security requirements engineering approach for trustworthy healthcare software development

**DOI:** 10.1186/s12911-020-01209-8

**Published:** 2020-09-18

**Authors:** Md Tarique Jamal Ansari, Fahad Ahmed Al-Zahrani, Dhirendra Pandey, Alka Agrawal

**Affiliations:** 1 Department of Information Technology, Babasaheb Bhimrao Ambedkar University, Lucknow, Uttar Pradesh India; 2 Department of Computer Engineering, College of Computer and Information Systems, Umm Al-Qura University, Mecca, Saudi Arabia

**Keywords:** Security requirements, Software security, Healthcare application, Quality software development, Fuzzy TOPSIS, Risk analysis

## Abstract

**Background:**

Today’s healthcare organizations want to implement secure and quality healthcare software as cyber-security is a significant risk factor for healthcare data. Considering security requirements during trustworthy healthcare software development process is an essential part of the quality software development. There are several Security Requirements Engineering (SRE) methodologies, framework, process, standards available today. Unfortunately, there is still a necessity to improve these security requirements engineering approaches. Determining the most suitable security requirements engineering method for trustworthy healthcare software development is a challenging process. This study is aimed to present security experts’ perspective on the relative importance of the criteria for selecting effective SRE method by utilizing the multi-criteria decision making methods.

**Methods:**

The study was planned and conducted to identify the most appropriate SRE approach for quality and trustworthy software development based on the security expert’s knowledge and experience. The hierarchical model was evaluated by using fuzzy TOPSIS model. Effective SRE selection criteria were compared in pairs. 25 security experts were asked to response the pairwise criteria comparison form.

**Results:**

The impact of the recognized selection criteria for effective security requirements engineering approaches has been evaluated quantitatively. For each of the 25 participants, comparison matrixes were formed based on the scores of their responses in the form. The consistency ratios (CR) were found to be smaller than 10% (CR = 9.1% < 10%). According to pairwise comparisons result; with a 0.842 closeness coefficient (Ci), STORE methodology is the most effective security requirements engineering approach for trustworthy healthcare software development.

**Conclusions:**

The findings of this research study demonstrate various factors in the decision-making process for the selection of a reliable method for security requirements engineering. This is a significant study that uses multi-criteria decision-making tools, specifically fuzzy TOPSIS, which used to evaluate different SRE methods for secure and trustworthy healthcare application development.

## Background

Today’s Information Technology (IT) has had a massive impact on a number of areas of society. We live in an era of IT in which technological resources such as software, hardware, and sensors are becoming an essential accessory in our daily lives [1, 2]. As we are increasingly dependent on software in our day-to-day life, therefore this technological dependency is a growing demand for secure software. Traditionally, software vendors have focused on improving the quality of software code to improve software security and quality [3]. Currently, security and privacy has become an emerging subject than ever between many healthcare organization, researchers, IT professionals, primarily due to the recent increase of Ransomware attacks around the world, and moreover due to the increasing amount of data and the exponential growth involved with the network of systems and technologies that produces and manipulates it. Unfortunately, attackers know the importance of data in patient medicinal records, rendering healthcare the biggest priority for cyber threats on ransomware comparative to any other sector.

The security violation for a healthcare software product causes enormous fatalities. For this reason, it is necessary to develop such a security-critical software system in the best conceivable way. It does not inevitably mean absolute security, however a reasonable high-security level in relation to the given limitations. In recent years, literature has offered a number of security and privacy requirements engineering methods that assist the software system designers and developers to implement security and privacy concerns presented in the traditional development model. Several methods deliberate security or privacy requirements independently, however, some other approaches consider privacy as a subset of security [4]. Software security requirements have become an important part of the overall requirements analysis process during the software development process.

**Fig. 1:**
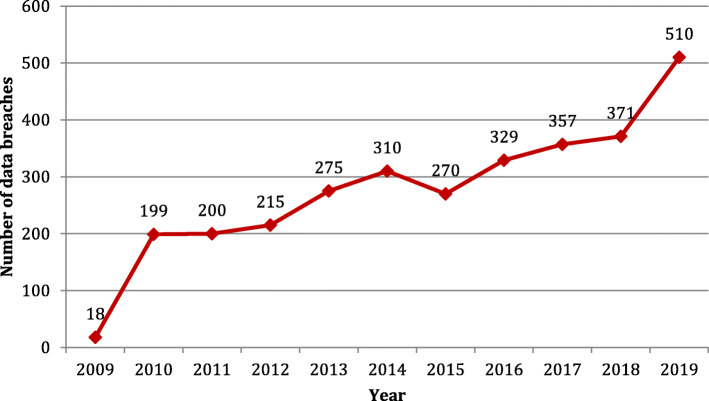
Year-wise healthcare data breaches (Source: HIPAA Journal)

In the United States in 1996 the Health Insurance Portability and Accountability Act, known as HIPAA, was enacted. The law sets standards for data security as well as privacy to safeguard patient records. HIPAA compliance has now become an important consideration in the healthcare industry for software engineers in recent times, as several high-profile data attacks have exposed millions of medical information nationally [5]. According to HIPAA Journal, there were 3054 data breaches in the healthcare industry affecting over 500 documents between 2009 and 2019. All these violations resulted in the destruction, theft, disclosure, or unauthorized release of 230,954,151 data in healthcare organizations. That is comparable to much more than 69.78% of the United States population. Data breaches of healthcare information were confirmed at a frequency of 1.4 per day in 2019 [6]. The following Fig. 1 shows the year-wise number of healthcare data breaches.

Software security is the fastest growing paradigm in the IT security field [7, 8]. According to the recent IDC Worldwide Semiannual Security Spending Guide, expenditure on security hardware, services, and software in Asia/Pacific is expected to reach USD 16.4 billion in 2019, an increase of 20.01% over the previous year. Further, the IDC assumes investment in software security-related products and services to grow at a five-year compound annual growth rate (CAGR) of 20.1% over the forecast period (2018–23) and reach USD 34 billion by 2023 [9].

Security requirements are one of the most important parts of all non-functional requirements. Negotiation with software security requirements during software development may result in disastrous failure of the software product affecting enormous damage of valuable assets. Special attention should be given to the security requirements of the software product during the software development as a software system does not exist physically.

The introduction of electronic health records raises a variety of problems related to protection and privacy that need addressing. Cyber-security in the healthcare sector has been one of the major challenges. Recent initiatives to digitize various dimensions of healthcare, the transition to electronic health, would have a huge effect on healthcare sector growth. Healthcare records and information are distributed across networks, which mean they are sensitive and vulnerable to a range of security breaches. The possible threats for healthcare breaches may include medical staff, malware or phishing attacks, healthcare suppliers, electronic health equipment, unauthorized access, etc. [5]. There are a variety of reasons for the targeted healthcare records. Several flaws in healthcare technology are available that can damage confidential healthcare data. Electronic medical records with other sensitive information can easily fall into fraudulent hands, despite proper monitoring. Huge amounts of confidential data and inadequate protection appear to be key factors, however the high value and reliability of the information is actually what encourages most attackers. Healthcare includes the highest proportion of weak authentication implementations and the closest frequency of data leakage. Such data points are also problematic considering the amount of healthcare services that interact with confidential patient data.

Obviously, the biggest challenge is to avoid improper access or misuse of the medical records. The next challenge is whether any permitted access is traceable. This in effect includes procedures for safe identification of individuals including doctors, patients, labs, etc. Eventually, documents in a censored or anonymized form ought to be made accessible to third parties. The rising threat to healthcare and patient’s confidential data enables application developers to become more rigorous in designing successful security requirements to make trustworthy healthcare web applications. For many healthcare application developers, security requirements are a major concern, and some of those have vital role in the proper organizational goals of the software development will try to incorporate security into the application development process as soon as possible.

**Fig. 2:**
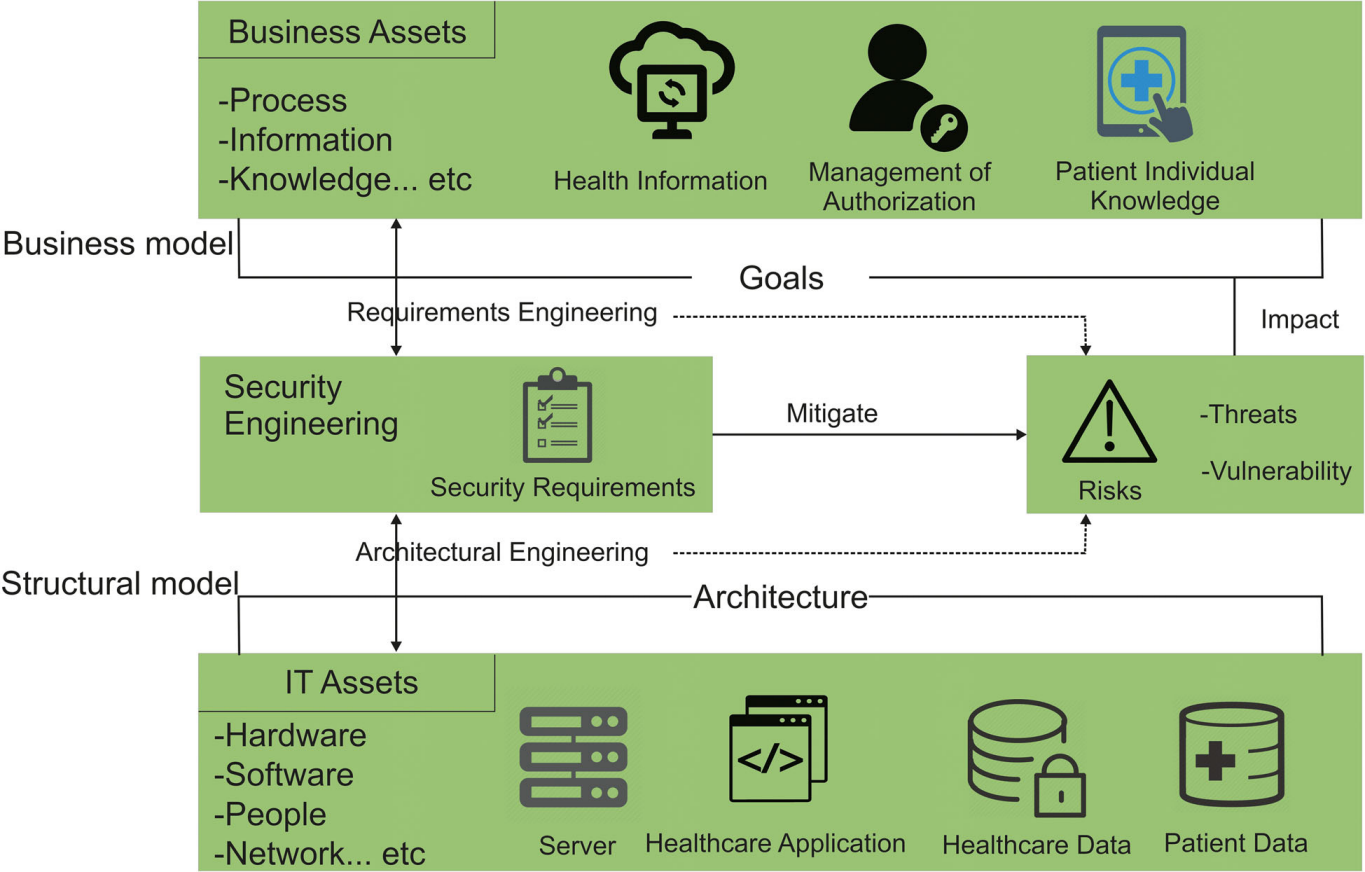
Security requirements engineering for trustworthy healthcare system

The identification of threats is significant and it facilitates the development of realistic and relevant security requirements. Implementations for healthcare services should be capable of storing and handle claims for refund. In addition, the database would be open to enable access to information about patient treatment in the event of immediate necessity. There’s really common understanding between many practitioners and researchers that security effective and efficient security requirements elicitation is important. Therefore the creation of healthcare applications requires the integration of functional, non-functional requirements and also architectural engineering practices [10]. One challenge to resolve is the significant advancements in the market needs, including during the creation of healthcare applications. Holding the emphasis on business properties, though, which tend to be much more constant, is an incentive for IT system growth to be better matched with the company. For a healthcare software application, assets are something that are confidential and has financial value to the organization, and that this is central to achieving its strategic goals. It is important to keep them protected. Figure 2 illustrates various types of assets for the healthcare application system. Business assets are patient confidential data, administration of healthcare authorizations, and patient’s individual records in healthcare industry, while the IT asset includes different hardware, software, associated stakeholders and networks. A web based healthcare application should properly incorporate all the effective security requirements in order to deliver trustworthy healthcare application software.

We notice that different security requirements engineering techniques are available for the elicitation of security requirements in order to develop a quality and trustworthy healthcare software system. However, the selection of the most appropriate SRE approach for trustworthy healthcare software development is a challenging task. The objective of this paper is to identify the different criteria for the analysis of different security requirements engineering approaches. Further, we select the ISO 27005 standard [8] criteria for the selection of effective security requirements engineering approach. We evaluate each criterion by itself or in comparison with other requirements, while ignoring the relationships existing between them, and also without regard to the effect of others on this priority value. In this paper, the researchers formally review the different security requirements engineering approaches and identify five best approaches as alternatives for comparative analysis. The main purpose of prioritizing existing security requirements engineering approaches is to help in quality and trustworthy software development. In order to improve decision making and to achieve this, it is necessary to establish a process adapted to requirements that take into account these relationships, to help provide consistency to the prioritization done. For that, before assigning a final value of priority to each criterion it is important to consider the operational significance of those criteria in the context of effective security requirements engineering with which it is in interdependency. In this paper we use the principle of pair-wise comparisons of fuzzy TOPSIS [11] method that is deliberated as the most helpful method, to help to accomplish the best decisions conceivable and to clearly present the rationality of the decision made about prioritization.

### Security requirements engineering

Security requirements engineering is an area of software engineering, which comprises security, safety, risk, vulnerabilities, and mitigation mechanisms. Security requirements engineering has over the years proven to be a challenging task. This is especially the case because pinpointing what security requirements are having been difficult. Despite all these challenges, the demand for developing security requirements elicitation methods for the changing requirements of networked environments is great. Mellado et al. [12] describe software security engineering as a practice through which to address software security issues in a systematic manner, is known to be a very important part of the software development process for the achievement of secure software systems. According to Devanbu & Stubblebine security requirement is considered as a manifestation of a high-level organizational policy into the detailed requirements of a specific system [13]. Lee et al. [14] draw attention to the significance of considering security requirements in the development life cycle, but do not define them. After analyzing the existing literature and best practices in the area of software security engineering, the authors have defined the security requirements engineering as:

“Security requirements engineering is a process of generating prerequisite non-functional requirements that stipulate a compulsory amount of system quality to prevent the adversary’s attack. It should be considered early during the software development process to deliver a trustworthy software product.”

Software security engineering procedure should include the use of repeatable and organized processes to guarantee that the set of requirements found is complete, reliable, easy to recognize and analyzable by the different participants involved in the software development process [15]. Security needs to be considered as a quality constraint in all the phases of software development process [16]. To develop a security-critical software system [17] many security requirement frameworks have been developed by different authors. Some of the famous security requirements engineering approaches are STORE [2], MOSRE [18], SREF [19], SREP [20], SQUARE [21]. Security requirements engineering is an important activity since bad security requirements can lead to ineffective security or worth security holes [22]. The following section briefly discusses each security requirements engineering approach.

### SREP

Mellado et al. proposed Security Requirements Engineering Process (SREP) [20] is a method that focuses on the Common Criteria security assessment standard [23] and is considered the principle of reuse. It deals systematically and intuitively with the security requirements. This approach offers a catalog of security resource management and synchronizes the Common Criteria into application development life cycle, in order to unify the notions of requirements engineering as well as security engineering. Several definitions and methods have been used in consideration of this method: a catalog of security tools (with properties, threats, specifications, etc.), cases of abuse, threat / attack trees, including cases of security uses. SREP was designed based on an understanding of the ISO / IEC 27002 standard [20].

SREP approach includes the following steps:

I. Agree on Definitions
II. Identify Vulnerable &/or Critical Assets
III. Identify Security Objectives & Dependencies
IV. Identify Threats & Develop Artifacts
V. Risk Assessment
VI. Elicit Security Requirements
VII. Categorize & Prioritize Requirements
VIII. Requirement Inspection
IX. Repository Improvement

### SQUARE

SQUARE (Security Quality Requirements Engineering) [21] developed by Carnegie Mellon University, It is a 9-step method aimed at categorizing and prioritizing the criteria for protection. This method provides a way to solicit, categorize, and prioritize security specifications for software applications. This methodology focuses on building features into the early stages of a software development lifecycle. It may also be effective to document and analyse the safety aspects of drafted applications and future changes may be guided and improvements to those structures. Every step is designated with inputs, outputs, members and procedures:

I. Agree on definitions
II. Identify security goals
III. Develop Artifacts to support security requirements definition
IV. Perform risk assessment
V. Select elicitation techniques
VI. Elicit security requirements
VII. Categorize requirements
VIII. Prioritize requirements
IX. Requirements inspection

The SQUARE method had been modified to handle privacy (P-SQUARE) as well as acquisition (A-SQUARE) explicitly.

### STORE

Ansari et al. developed Security Threat Oriented Requirements Engineering Methodology (STORE) Methodology which is a ten-step security threat centric security requirements engineering methodology [2]. It provides methodological standards for organizational security systems with the help of standard system platforms as well as interfaces in the perspective of increased possible key infrastructure safety risks. The following section lists all the steps of STORE methodology as well as describing the functionality of each step.

I. Identify System Goals
II. Identify and Prioritize Stakeholders
III. Agreed upon Goals
IV. Asset Identification
V. Security Attack Analysis
  Point of Attack (PoA)
  Point of Belief (PoB)
  Point of Conjecture (PoC)
  Point of Dependency (PoD)
VI. Threat Identification and Categorization
VII. Risk Evaluation and Prioritization
VIII. Security Requirements Elicitation
IX. Security Requirements Validation
X. Security Requirements Specification Document

### MOSRE

The Model Oriented Security Requirements Engineering approach [18] seeks to use different models such as application use cases, a misuse case etc. to enable quality and safety and requires analysis. It is designed to refer to web based application development. The peculiarity of MOSRE is that it includes the verification of objectives for whole system, the identification and modeling of non-security requirements prior to actually addressing the security requirements. It is therefore a technique that can be implemented to the entire phase of requirements engineering, with special emphasis on safety. Different stages of MOSRE framework are:

I. Inception: Recognize web app goals, stakeholders as well as assets
II. Elicitation
III. Elaboration: Produce structural analysis models
IV. Negotiation and validation of requirements

### SREF

Haley et al. proposed Security Requirements Engineering Framework [19] which is a combination of engineering requirements as well as security requirements. This is recursive as it reverts back and forward between design and technical specifications. The SREF approach provides following four steps:

I. Identify functional requirements
II. Identify security goals

- Identify assets
- Generate threat description
- Apply management principles (separation of duties, functions, ..)

III. Identify security requirements: Constraints on some or all of the security objectives. The safety requirements are descriptively represented.
IV. Construct satisfaction arguments: represent that the application is capable of meeting security requirements.

## Methods

To make good decisions we must well prioritize the recognized ISO 27005 standard criteria for effective security requirement engineering selection, and so it is important to take the consideration of all 25 security experts into account when prioritizing. In our prioritization process we use the technique of pairwise comparison of different criteria through fuzzy TOPSIS. In order to selection of best SRE approach; we need to compare pairs of these criteria.

### Criteria and alternatives selection

**Fig. 3:**
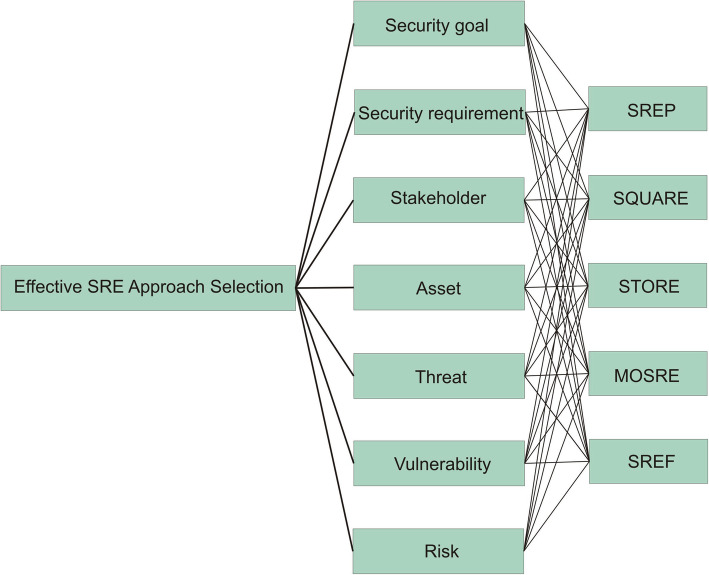
Hierarchical model of criteria and alternatives

The first step in this process is the establishment of a hierarchy model. The hierarchy model is composed of the seven criterion group Security goal, Security requirement, Stakeholder, Asset, Threat, Vulnerability, Risk. After the hierarchy has been established, the criteria must be evaluated in pairs so as to determine the relative importance between them and their relative weight to the global goal. The study was planned and conducted to comparatively evaluate the different criteria which are considered by the software developer during the selection of effective security requirements engineering approach. A survey form was prepared to determine the prioritization of the characteristics incorporated when choosing effective security requirements engineering approach for the development of trustworthy healthcare software system. This form was given to 25 security experts to collect their estimation on the pair-wise criteria comparisons and fuzzy TOPSIS model was created. A set of ISO 27005 standard criteria represents the balanced hierarchical structure consisting of the seven main criteria and five alternatives incorporating the objectives and criteria when choosing the effective alternative as a security requirements engineering approach. Some of the popular SRE approaches which are used in this study are SREP, SQUARE, STORE, MOSRE and SREF which are represented by A1, A2, A3, A4, and A5 respectively. The Fig. 3 shows the hierarchy representation of different criteria and alternatives.

We mailed pairwise questionnaire form based on seven points scale to each of the security experts. The security experts were given a questionnaire that contained a pairwise comparison sheet. The members consisted of 25 security experts who were serving in the different software organizations and who had experience exceeding 15 years having deep knowledge about security requirements engineering process during software development. All security experts were security domain experts who practice different security requirements in their working places to build quality software products and have valuable knowledge about the different modern threats and software attack mechanism. They responded about their satisfaction with their choices. Further the collected data analyzed separately for each security expert. The following Table 1 shows the detailed description of each criterion.

**Table 1:** The ISO 27005 standard criteria for effective SRE approach selection

Criteria	Description
Security goal (C1)	Security goals clearly state what the software system must avoid and not how that preventative measures should be accomplished.
Security requirement (C2)	Security requirements are implications of software system threats that can be obtained only from design process. Security requirements quite precisely reflect safety objectives.
Stakeholder (C3)	A stakeholder is a person, an organization or a community with an interest with the under development software system. A Stakeholder perspective defines a specific stakeholder’s requirements. The stakeholders can show various kinds of requirements.
Asset (C4)	Software asset would be any process / service that a corporation uses as part of the economic operations. For companies, monitoring and managing such assets is essential, as they may involve regulatory risks, threats to brand equity and even existence.
Threat (C5)	Threats to software system are harmful elements of computer programs and programs that can potentially harm your computer or capture personal and financial information.
Vulnerability (C6)	Vulnerability may consider as software system defect that can consider leaving it open to manipulation. Vulnerability may also correspond to any kind of deficiency in a software system on its own, in a set of processes, or even anything which leaves the security and privacy of data at risk.
Risk (C7)	Risk is a failure prediction; a possible issue that might or might not arise in the future. It is usually limited by inadequate of information, regulation or time. It is possibility of experiencing from failure in software development life cycle.

### Fuzzy TOPSIS method

Many multi-criteria-decision-making (MCDM) methods were developed in order to rank alternatives differently. Although evaluations of alternatives offered by MCDM methods may sometimes be in contract, there are circumstances where distinct MCDM methods produce very different recommendations [24–26]. A finite number of possible outcomes are selected, prioritized and ranked by experts. Because there are several techniques involved, Hwang and Yoon also include taxonomy to identify the methods as: assessment metrics from experts, essential information attributes, and a big class of methods. Even the description gives us a simple path to learn MCDM approaches [11]. They introduced Technique for Order of Preference by Similarity to Ideal Solution (TOPSIS) which defines an instrument called correlation to the ideal-positive solution as well as disconnection from the ideal-negative. The technique then prefers an alternative with highest correlation to the ideal-positive solution. They presented the TOPSIS method built on the concept that even a significant short distance through an ideal solution ought to be the best option available.

Several researches are available wherein the authors used the fuzzy TOPSIS method to develop effective decisions. Ashrafzadeh et al. [27] presented a Fuzzy TOPSIS method to multi-criteria decision making for choosing warehouse location under limited or imperfect data. They first defined the different selection criteria for the warehouse location and afterwards presented the alternative approaches against all the selected criteria with the help of domain experts with linguistic scale evaluations. Fuzzy TOPSIS is often used to produce selection of the best alternatives for aggregate scores. They also demonstrate a successful implementation of fuzzy TOPSIS to something like a true issue of selecting a big corporation’s warehouse location in Iran. Sevkli et al. [28] used the fuzzy TOPSIS method to select suppliers at a Turkish manufacturing industry. They applied this method to assess the performance of alternatives on the shopping site and rank the primary importance appropriately for each other. TOPSIS considers a MADM issue as a geometric structure with m points in n-dimensional space, with m alternatives. The approach is based on the principle that the selected alternative should be the quickest range from the ideal-positive solution as well as the longest range from the ideal-negative.

In this method if each characteristic takes on asymptotically raising or lowering variation, then maybe an ideal solution can be easily defined. That solution consists of all possible to achieve best attributes, since the worst solution consists of all attainable worst attribute values. Assumed a decision-making issue with multiple criteria has n alternatives, A1, A2, ..., An and m criteria, C1, C2, ..., Cm. Each alternative is assessed against the criteria of m. All the values/ratings are allocated to alternatives regarding decision matrix represented by X(x_ij_)_m × n_. Let W = (w_1_, w_2_, …, w_m_) be the weight vector of criteria, satisfying ${\sum}_{\mathrm{j}-1}^{\mathrm{m}}{\mathrm{w}}_{\mathrm{j}}=1.$

**Fig. 4:**
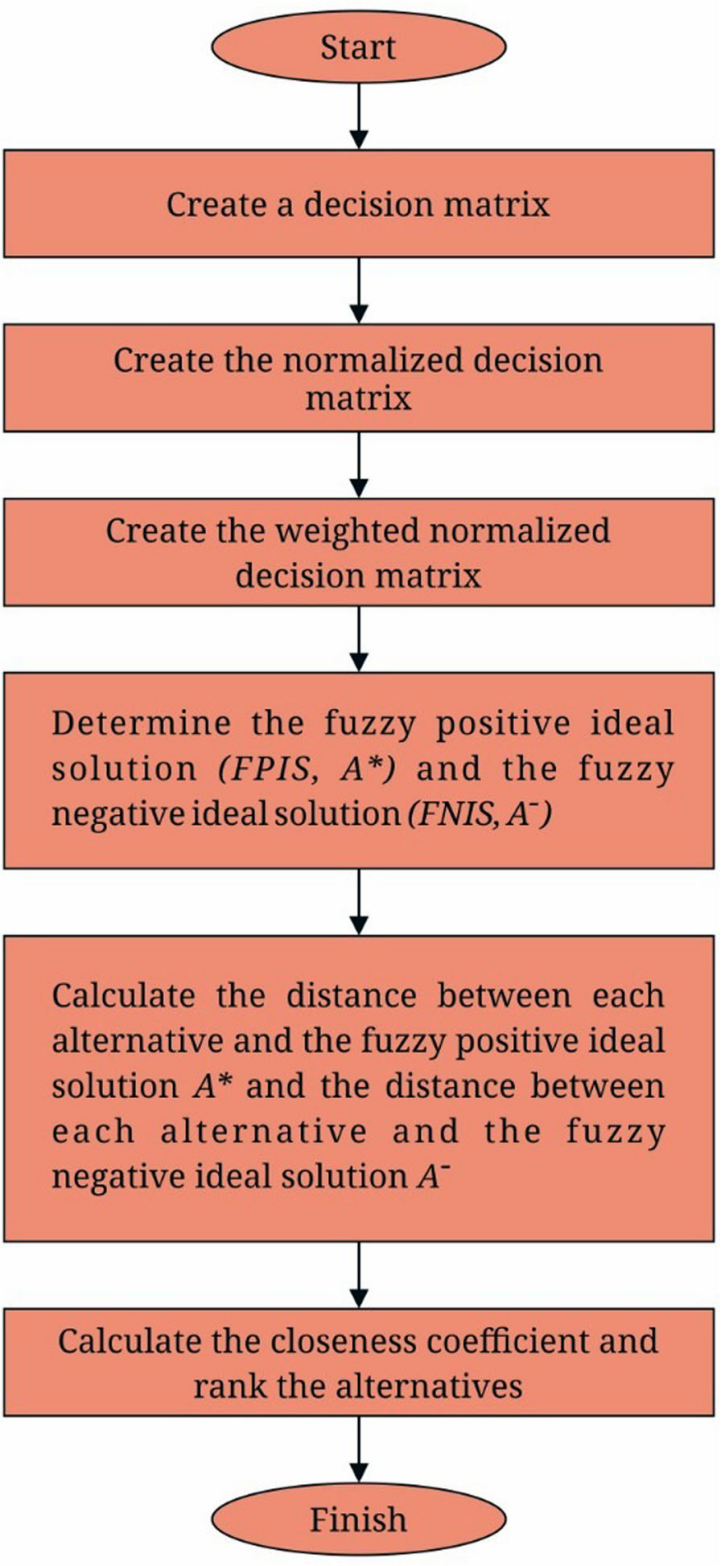
Flowchart of the fuzzy TOPSIS process

The fuzzy TOPSIS method comprises of the following steps as shown in Fig. 4.

### Step 1 create a decision matrix

In this research study there are 7 criteria and 4 alternatives which are rated and ranked according to the FUZZY TOPSIS system. The following Table 2 describes the characteristics of criterion as well as weight assigned with each criterion.

**Table 2:** Characteristics of Criteria

	Criteria	Type	Weight
1	C1	+	0.143,0.143,0.143
2	C2	+	0.143,0.143,0.143
3	C3	+	0.143,0.143,0.143
4	C4	+	0.143,0.143,0.143
5	C5	+	0.143,0.143,0.143
6	C6	+	0.143,0.143,0.143
7	C7	+	0.143,0.143,0.143

The Table 3 below shows the seven point fuzzy scale used in this research study.

**Table 3:** Fuzzy Scale

Code	Linguistic terms	L	M	U
1	Very low	0	0	1
2	Low	0	1	3
3	Moderately low	1	3	5
4	Moderate	3	5	7
5	Moderately high	5	7	9
6	High	7	9	10
7	Very high	9	10	10

### Step 2 create the normalized decision matrix

A normalized decision matrix can be estimated by the following relation based on the positive and negative ideal solutions:

$$ {\displaystyle \begin{array}{l}{\overset{\sim }{\mathrm{r}}}_{\mathrm{i}\mathrm{j}}=\left(\frac{{\mathrm{a}}_{\mathrm{i}\mathrm{j}}}{{\mathrm{c}}_{\mathrm{j}}^{\ast }},\frac{{\mathrm{b}}_{\mathrm{i}\mathrm{j}}}{{\mathrm{c}}_{\mathrm{j}}^{\ast }},\frac{{\mathrm{c}}_{\mathrm{i}\mathrm{j}}}{{\mathrm{c}}_{\mathrm{j}}^{\ast }}\right);{\mathrm{c}}_{\mathrm{j}}^{\ast }={\max}_{\mathrm{i}}\ {\mathrm{c}}_{\mathrm{i}\mathrm{j}};\mathrm{Positive}\ \mathrm{ideal}\ \mathrm{solution}\\ {}{\overset{\sim }{\mathrm{r}}}_{\mathrm{i}\mathrm{j}}=\left(\frac{{\mathrm{a}}_{\mathrm{j}}^{-}}{{\mathrm{c}}_{\mathrm{i}\mathrm{j}}},\frac{{\mathrm{a}}_{\mathrm{j}}^{-}}{{\mathrm{b}}_{\mathrm{i}\mathrm{j}}},\frac{{\mathrm{a}}_{\mathrm{j}}^{-}}{{\mathrm{a}}_{\mathrm{i}\mathrm{j}}}\right);{\mathrm{a}}_{\mathrm{j}}^{-}={\min}_{\mathrm{i}}\ {\mathrm{a}}_{\mathrm{i}\mathrm{j}};\mathrm{Negative}\ \mathrm{ideal}\ \mathrm{solution}\end{array}} $$

### Step 3 create the weighted normalized decision matrix

Depending on different weights within each criterion, as per the following formula, the weighted normalized decision matrix can be determined by calculating the weight of each criterion in the standard fuzzy decision matrix.

$$ {\overset{\sim }{\mathrm{v}}}_{\mathrm{ij}}={\overset{\sim }{\mathrm{r}}}_{\mathrm{ij}}.{\overset{\sim }{\mathrm{w}}}_{\mathrm{ij}} $$

Where $\tilde{w}_{ij}$ represents weight of criterion c_j_

### Step 4 determine the fuzzy positive-ideal solution (FPIS) a* and fuzzy negative-ideal solution (FNIS) A-

The FPIS and FNIS of the alternatives can be defined as follows:

$$ {\displaystyle \begin{array}{c}{\mathrm{A}}^{\ast }=\left\{{\overset{\sim }{\mathrm{v}}}_1^{\ast },{\overset{\sim }{\mathrm{v}}}_2^{\ast },\dots, {\overset{\sim }{\mathrm{v}}}_{\mathrm{n}}^{\ast}\right\}=\left\{\left(\underset{\mathrm{j}}{\max }{\mathrm{v}}_{\mathrm{ij}}|\mathrm{i}\in \mathrm{B}\right),\left(\underset{\mathrm{j}}{\min }{\mathrm{v}}_{\mathrm{ij}}|\mathrm{i}\in \mathrm{C}\right)\right\}\\ {}{\mathrm{A}}^{-}=\left\{{\overset{\sim }{\mathrm{v}}}_1^{-},{\overset{\sim }{\mathrm{v}}}_2^{-},\dots, {\overset{\sim }{\mathrm{v}}}_{\mathrm{n}}^{-}\right\}=\left\{\left(\underset{\mathrm{j}}{\min }{\mathrm{v}}_{\mathrm{ij}}|\mathrm{i}\in \mathrm{B}\right),\left(\underset{\mathrm{j}}{\max }{\mathrm{v}}_{\mathrm{ij}}|\mathrm{i}\in \mathrm{C}\right)\right\}\end{array}} $$

Where $\tilde{v}_{i}^{\ast }$ is the max value of i for all the alternatives and ${\overset{\sim }{v}}_1^{-}$ is the min value of i for all the alternatives. *B* and *C* represent the positive and negative ideal solutions, respectively.

### Step 5 calculate the distance between each alternative and the fuzzy positive ideal solution a+ and the distance between each alternative and the fuzzy negative ideal solution

The distance between each alternative and FPIS and the distance between each alternative and FNIS are respectively calculated as follows:

$$ {\displaystyle \begin{array}{l}{\mathrm{S}}_{\mathrm{i}}^{\ast }={\sum}_{\mathrm{j}=1}^{\mathrm{n}}\mathrm{d}\left({\overset{\sim }{\mathrm{v}}}_{\mathrm{i}\mathrm{j}},{\overset{\sim }{\mathrm{v}}}_{\mathrm{j}}^{\ast}\right)\mathrm{i}=1,2,\dots, \mathrm{m}\\ {}{\mathrm{S}}_{\mathrm{i}}^{-}={\sum}_{\mathrm{j}=\mathrm{i}}^{\mathrm{n}}\mathrm{d}\left({\overset{\sim }{\mathrm{v}}}_{\mathrm{i}\mathrm{j}},{\overset{\sim }{\mathrm{v}}}_{\mathrm{j}}^{-}\right)\ \mathrm{i}=1,2,\dots, \mathrm{m}\end{array}} $$

d is the distance between two fuzzy numbers, when given two triangular fuzzy numbers (*a*_1_, *b*_1_, *c*_1_) and (*a*_2_, *b*_2_, *c*_2_), e distance between the two can be calculated as follows:

$$ {d}_v\left({\overset{\sim }{M}}_1,{\overset{\sim }{M}}_2\right)=\sqrt{\frac{1}{3}\left[{\left({a}_1-{a}_2\right)}^2+{\left({b}_1-{b}_2\right)}^2+{\left({c}_1-{c}_2\right)}^2\right]} $$

Note that $d\left(\tilde{v}_{ij},\tilde{v}_{j}^{\ast}\right)$ and $d\left(\tilde{v}_{ij},\tilde{v}_{j}^{-}\right)$ are crisp numbers.

### Step 6 calculate the closeness coefficient and rank the alternatives

The closeness coefficient of each alternative can be calculated as follows:

$$ {\mathrm{CC}}_{\mathrm{i}}=\frac{{\mathrm{S}}_{\mathrm{i}}^{-}}{{\mathrm{S}}_{\mathrm{i}}^{+}+{\mathrm{S}}_{\mathrm{i}}^{-}} $$

## Result

This section provides a systematic and accurate description of the observational data, their viewpoint as well as the findings to be drawn from the experiment. The alternatives in terms of various criteria are evaluated and the results of the decision matrix are shown as follows. The matrix below represents the arithmetic mean of all experts when multiple experts participate in the evaluation. The following Table 4 shows the decision matrix with respect to criteria and alternatives.

**Table 4:** Decision matrix

	C1	C2	C3	C4	C5	C6	C7
A1	3.480,5.480,7.440	4.680,6.680,8.520	5.080,7.000,8.520	4.920,6.880,8.560	5.080,7.000,8.480	5.800,7.680,9.120	5.240,7.160,8.720
A2	4.760,6.760,8.560	5.320,7.280,8.880	4.680,6.680,8.480	5.560,7.560,9.080	5.560,7.440,8.840	5.160,7.040,8.560	5.000,7.000,8.600
A3	4.840,6.800,8.480	5.480,7.440,9.000	5.320,7.280,8.920	5.480,7.400,8.920	5.400,7.280,8.840	5.120,7.000,8.520	5.240,7.160,8.800
A4	4.600,6.600,8.320	5.160,7.080,8.640	4.760,6.720,8.400	4.680,6.640,8.360	4.920,6.800,8.440	4.680,6.680,8.480	4.520,6.520,8.240
A5	4.680,6.680,8.520	5.000,6.960,8.560	5.160,7.120,8.760	4.840,6.800,8.520	4.680,6.680,8.480	4.520,6.520,8.320	4.360,6.360,8.200

Further the following Table 5 shows the normalized decision matrix.

**Table 5:** A normalized decision matrix

	C1	C2	C3	C4	C5	C6	C7
A1	0.407,0.640,0.869	0.520,0.742,0.947	0.570,0.785,0.955	0.542,0.758,0.943	0.575,0.792,0.959	0.636,0.842,1.000	0.595,0.814,0.991
A2	0.556,0.790,1.000	0.591,0.809,0.987	0.525,0.749,0.951	0.612,0.833,1.000	0.629,0.842,1.000	0.566,0.772,0.939	0.568,0.795,0.977
A3	0.565,0.794,0.991	0.609,0.827,1.000	0.596,0.816,1.000	0.604,0.815,0.982	0.611,0.824,1.000	0.561,0.768,0.934	0.595,0.814,1.000
A4	0.537,0.771,0.972	0.573,0.787,0.960	0.534,0.753,0.942	0.515,0.731,0.921	0.557,0.769,0.955	0.513,0.732,0.930	0.514,0.741,0.936
A5	0.547,0.780,0.995	0.556,0.773,0.951	0.578,0.798,0.982	0.533,0.749,0.938	0.529,0.756,0.959	0.496,0.715,0.912	0.495,0.723,0.932

The following Table 6 demonstrates the weighted normalized decision matrix

**Table 6:** The weighted normalized decision matrix

	C1	C2	C3	C4	C5	C6	C7
A1	0.058,0.092,0.124	0.074,0.106,0.135	0.081,0.112,0.137	0.077,0.108,0.135	0.082,0.113,0.137	0.091,0.120,0.143	0.085,0.116,0.142
A2	0.080,0.113,0.143	0.085,0.116,0.141	0.075,0.107,0.136	0.088,0.119,0.143	0.090,0.120,0.143	0.081,0.110,0.134	0.081,0.114,0.140
A3	0.081,0.114,0.142	0.087,0.118,0.143	0.085,0.117,0.143	0.086,0.117,0.140	0.087,0.118,0.143	0.080,0.110,0.134	0.085,0.116,0.143
A4	0.077,0.110,0.139	0.082,0.112,0.137	0.076,0.108,0.135	0.074,0.105,0.132	0.080,0.110,0.137	0.073,0.105,0.133	0.073,0.106,0.134
A5	0.078,0.112,0.142	0.079,0.111,0.136	0.083,0.114,0.140	0.076,0.107,0.134	0.076,0.108,0.137	0.071,0.102,0.130	0.071,0.103,0.133

**Table 7:** The positive and negative ideal solutions

	Positive ideal	Negative ideal
C1	0.081,0.114,0.143	0.058,0.092,0.124
C2	0.087,0.118,0.143	0.074,0.106,0.135
C3	0.085,0.117,0.143	0.075,0.107,0.135
C4	0.088,0.119,0.143	0.074,0.105,0.132
C5	0.090,0.120,0.143	0.076,0.108,0.137
C6	0.091,0.120,0.143	0.071,0.102,0.130
C7	0.085,0.116,0.143	0.071,0.103,0.133

The positive and negative ideal solutions are shown in the Table 7.

The Table 8 shows distance from positive and negative ideal solutions.

The best alternative is closest to the FPIS and farthest to the FNIS. The closeness coefficient of each alternative and the ranking order of it are shown in the Table 9.

**Fig. 5:**
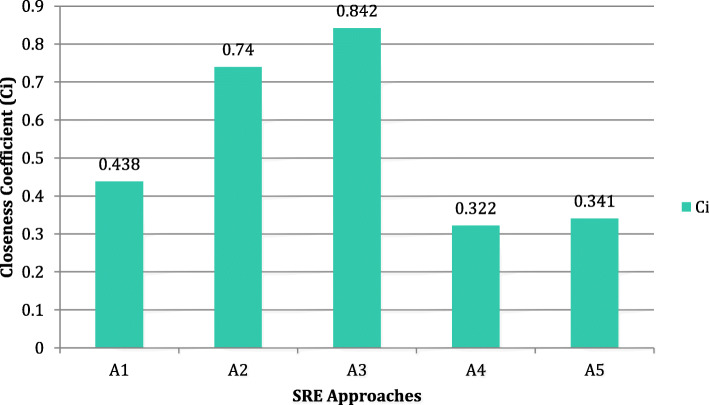
Closeness coefficient graph

The following Fig. 5 shows the closeness coefficient of each alternative based on the findings of this research study.

**Table 8:** Distance from positive and negative ideal solutions

	Distance from positive ideal	Distance from negative ideal
A1	0.055	0.043
A2	0.025	0.072
A3	0.015	0.082
A4	0.066	0.031
A5	0.064	0.033

**Table 9:** Closeness coefficient

	Ci	Rank
A1	0.438	3
A2	0.74	2
A3	0.842	1
A4	0.322	5
A5	0.341	4

The closeness coefficient (Ci) of different alternatives is estimated as 0.438, 0.74, 0.842, 0.322 and 0.341 for A1, A2, A3, A4 and A5 respectively. The findings show the A3 has highly effective and efficient security requirements engineering approach for trustworthy healthcare software development.

## Discussion

Today’s several malicious attempts are by technological flaws in healthcare applications such as SQL injection, session hijacking or cross-site scripting. Healthcare applications which are susceptible to such vulnerabilities have become a convenient target for cyber criminals and therefore most frequently lead to confidential information breaches. The confidentiality of patient’s data remains the most significant challenge to reach when contemplating the implementation of Healthcare Information Systems (HIS) in the healthcare sector. Recently, so many studies were performed to recognize potential healthcare security threats, as well as a variety of alternatives were suggested to facilitate security requirements for security-critical healthcare applications. In this study we undertake the powerful fuzzy TOPSIS technique for the selection of effective Security Requirements Engineering approach to assist the software developers in developing trustworthy healthcare software.

Based on the findings, it is clear that Security Threat Oriented Requirements Engineering (STORE) methodology is the most effective SRE approach based on the security experts’ selections. This undoubtedly shows that the recognition of threats is very much significant and every security requirement engineering approach should consider this criterion. In many existing security requirements engineering methods, the threat identification has played a significant role in eliciting effective security requirements for a software project. Prior to eliciting the effective security requirements of a healthcare software system, it is significant to identify all the possible threats to the software system. Assessing the threats to a software system assists software developers to build complete and accurate security requirements [2, 29].

Further risk analysis and stakeholder’s involvement in the security requirements engineering process is also important. Although considering stakeholder’s view in security requirement engineering is an important concern, but only some security requirements engineering approaches to address this concern. This doesn’t mean that it is impossible to consider the views of different stakeholders using other methods. However, most of the security requirements do not capture this issue in their various activities [30–33]. The security requirements engineering approach should propose steps to establish cooperation between different security concerns accepted by different stakeholders. All potential stakeholders must be incorporated during the security requirements engineering process. There are comprehensive security threat models but stakeholder’s identification is not emphasized. There is a need to provide steps for considering the security interests of all the stakeholders of the software system and also involves the effective mechanism for threat agent identification and risk analysis for easy, complete, and well-organized security requirements engineering.

After the criteria were prioritized accordingly, the model used in this study enabled us to analyses the main concept of the consistency of preferences made by the security experts. Furthermore, the main purpose of this study is to provide a model enabling security experts to make a more consistent decision for trustworthy healthcare software development. After the features of the security requirements engineering method became clear, this model can be used to predict effective security requirements engineering approach selection in the real world. This study determined the priority of alternatives that are considered in selecting an effective security requirements engineering method respective of criteria for trustworthy healthcare software development. These criteria highlight the prioritized SRE approaches to which a software developer should pay attention.

## Conclusions

Effective security requirements engineering approach selection decision is essential for the trustworthy healthcare software development. Determining among the many existing SRE methods is a challenging decision-making problem due to the fact that each approach has advantages as well as disadvantages. We implemented the results of a study on the application of fuzzy TOPSIS methodology. A set of ISO 27005 standard criteria identified based on the literature review and organized into a rational hierarchical structure consisting of the seven main criteria and five alternatives. The consistency ratios were less than 0.10 for all the 25 security experts in collected form responses. The research findings suggest that the STORE approach (with Ci value 0.842) is more effective than SQUARE (0.74), SREP (0.438), SREF (0.341), and MOSRE (0.322) in manipulating performance towards security requirements engineering approaches. Determining weights of essential motivation, purpose, and consciousness focus areas can help security decision-making and compliance with policy, and support design of effective security requirements engineering. However, these weights may in turn be affected by local organizational and educational factors. The presented fuzzy TOPSIS results in this paper can be used to select or design an effective security requirements engineering approach that may assist the software developers in developing a trustworthy healthcare software system. Several other fuzzy decision-making approaches are available like VIKOR, fuzzy ANP, PROMETHE, and many others can be used for future research, and their findings can be compared with the findings obtained in this study. The outcome discussed in this research may be used by the software professionals working in the clinical, education, and healthcare activities related to software development.

## Data Availability

All data generated or analysed during this study are included in this paper.

## Abbreviations

MCDM: Multi-criteria decision making
MADM: Multiple Attribute Decision-making
NIS: Negative ideal solution
PIS: Positive ideal solution
TOPSIS: Technique for order preference by similarity to ideal solution
SRE: Security Requirements Engineering
